# Draft genome sequence of Kei apple, an underutilized African tree crop

**DOI:** 10.1038/s41597-025-04414-0

**Published:** 2025-01-15

**Authors:** Robert Kariba, Bernice Waweru, Isaac Njaci, Linly Banda, Jenniffer W. Mwangi, Antso Harimanantsoa R, Samuel Muthemba, Jonathan Featherstone, Prasad Hendre, Ramni Jamnadass, Allen Van Deynze, Jean-Baka Domelevo Entfellner, Oluwaseyi Shorinola

**Affiliations:** 1 World Agroforestry, P.O Box 30677, Nairobi, 00100 Kenya; 2 International Livestock Research Institute, P.O. Box 30709, Nairobi, 00100 Kenya; 3 John Innes Centre, Norwich Research Park, Norwich, NR4 7UH UK; 4 School of Agriculture and Food Sustainability, The University of Queensland, Brisbane, QLD Australia; 5 Department of Horticulture and Landscape Architecture, Colorado State University, Colorado, USA; 6 School of Pure and Applied Sciences, Machakos University, P.O. Box 136, 90100 Machakos, Kenya; 7 Faculty of Science, University of Antananarivo, BP 906, Antananarivo, 101 Madagascar; 8 Agricultural Research Council, Biotechnology Platform, Pretoria, 0110 South Africa; 9 Department of Plant Sciences, University of California, Davis, CA 95616 USA; 10 School of Biosciences, University of Birmingham, Edgbaston, Birmingham, 15 2TT UK

**Keywords:** Conservation genomics, Agriculture

## Abstract

To address food and nutrition security in the face of burgeoning global populations and erratic climatic conditions there is a need to include nutrient dense, climatic resilient but neglected indigenous fruit trees in agrifood systems. Here we present the draft genome sequence of Kei Apple, *Dovyalis afra*, a neglected indigenous African fruit tree with untapped potential to contribute to nutrient security and improved livelihoods. Our long-read-based genome assembly comprises 440 Mbp sequence across 1190 contigs with a N50 and L50 of 13.3 Mbp and 11, respectively. We also annotated the genome and identified 27,449 protein-coding genes. Our genome assembly provides a valuable resource for unlocking the food security and nutraceutical potential of Kei apple.

## Background & Summary

Decades of intensive research on a few species have led to the development of high yielding varieties and agricultural systems focusing on major crops. These major crops, which are mostly carbohydrate-rich, cannot sufficiently provide the dietary diversity required for a healthy population. Diets rich in fruits and vegetables provide a varied source of dietary vitamins, minerals, antioxidants and fiber that not only support normal physiological function but also reduces disease risks^1–5^. Utilization of a diverse array of fruits particularly those that are underutilized, can contribute greatly to diversification, sustainability and affordability. These underutilized fruit crops typically exhibit genetic tolerance allowing them to survive and thrive under harsh conditions. Additionally, they often possess noteworthy nutritional and/or industrial qualities which render them useful for a variety of purposes^6^.

**Fig. 1:**
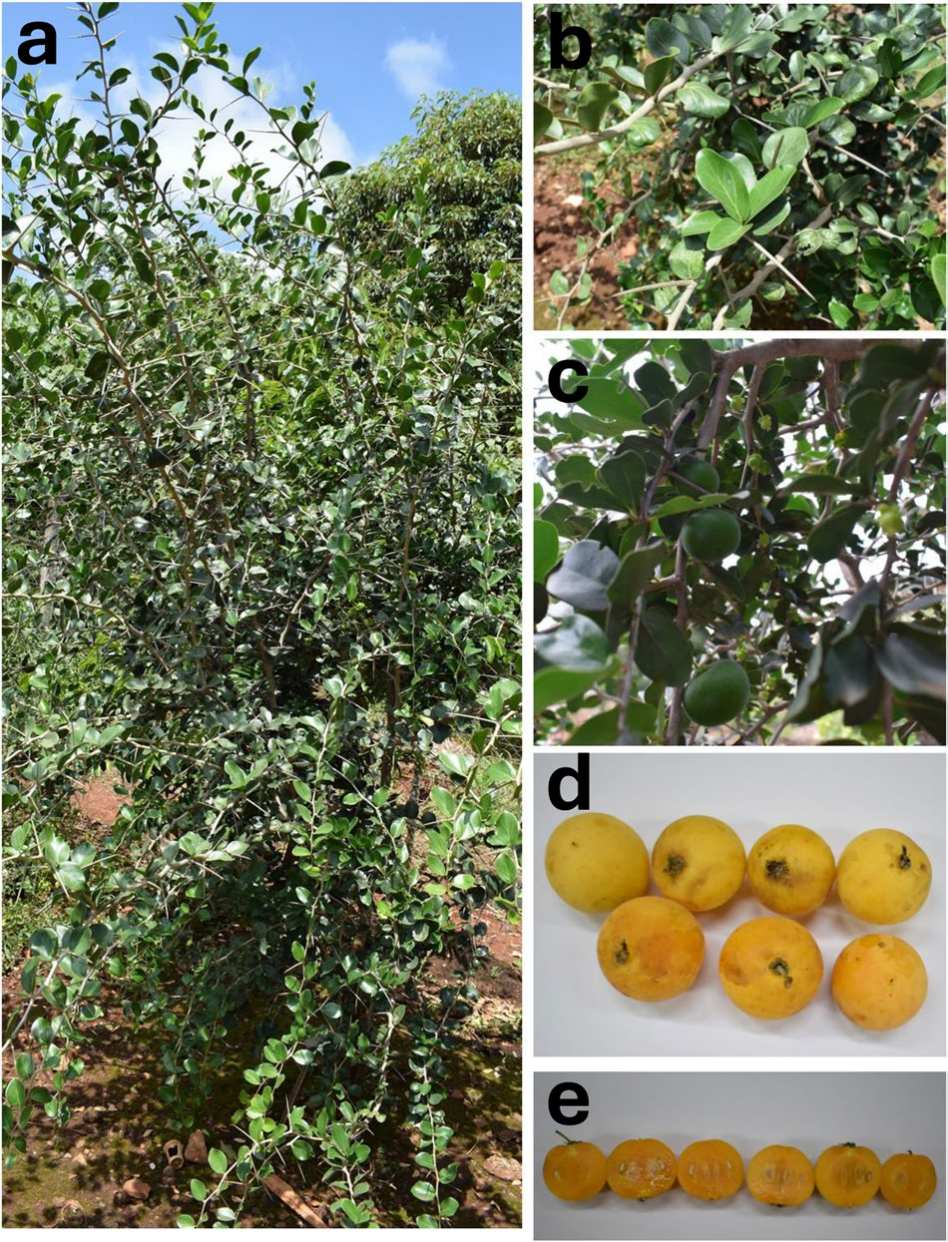
*Dovyalis afra* (Kei Apple). The sequenced Dovyalis plant (**a**) is shown with its leaves (**b**), unripe fruits (**c**), ripe fruits (**d**) and fruit section (**e**).

*Dovyalis afra* (Hook.f. & Harv.) Sim, commonly referred to as the Kei apple (hereafter referred to as Dovyalis, represents an underutilized indigenous African fruit tree belonging to the *Salicaceae* family (Fig. 1). Dovyalis’ fruit is nutritionally dense, providing substantial amounts of carbohydrates, crude fiber^7^, moderate levels of β-carotene (pro-vitamin A), elevated concentrations of ascorbic acid (vitamin C)^8^, and various minerals. Dovyalis is a notable source of phytochemicals and other bioactive compounds with known antioxidant and anticancer activities^8–10^, holding great potential for human health. All the plant parts have a history of use in traditional medicine to treat various disease conditions^11,12^. The fruit juice extract has shown effectiveness against bacteria and yeast growth, and the methanolic extract has reported activity against HIV^13^ and human coronavirus 229E^14^. The fruit possesses potential for processing or incorporation into various products such as ready to drink juices, wine, vinegar, jams and jellies^8^.

Intensified research and utilization of underutilized crops has the potential to significantly improve nutrition and combat hidden hunger especially in poor households in the rural, arid, and semiarid areas. In this study, we sequenced and assembled the genome of Dovyalis. This establishes a resource that will facilitate the exploration of the genetic potential and in the future, the genetic diversity within the species.

## Methods

### High molecular weight genomic DNA extraction

Fresh leaves were harvested from a *Dovyalis* *afra* tree (ICRAF 04990) maintained at World Agroforestry (ICRAF) Nairobi, Kenya, and immediately flash-frozen in liquid nitrogen. The frozen leaves were ground in liquid nitrogen in a mortar and pestle and high molecular weight (HMW) genomic DNA was extracted following the protocol provided by Oxford Nanopore Technologies (ONT) on “High Molecular Weight gDNA extraction from fever tree leaves (*Cinchona pubescens*)”, downloaded from the ONT community in June 2019^15^. Briefly, Carlson lysis buffer (100 mM Tris-HCl, pH 9.5, 2% CTAB, 1.4 M NaCl, 1%, PEG 8000, 20 mM EDTA) was used to lyse the cells, followed by chloroform phase-fractionation. The HMW genomic DNA was bound and eluted using the Qiagen Genomic Tips (Qiagen, Hilden, Germany). The HMW genomic DNA was cleaned using RNAse and Proteinase K. HMW genomic DNA longer than 10 Kbp was selected using a polyethylene glycol (PEG) and salt solution (9% PEG 8,000, 1 M NaCl, 10 mM Tris-HCl pH 8) according to Jones *et al*.^16^.

### DNA nanopore sequencing

Sequencing libraries were prepared according to the ONT SQK-LSK109 ligation sequencing kit (ONT, Oxford, England) protocol. A 1.5 μg aliquot of the HMW genomic DNA was used. Repair and 3′ adenylation was done using the NEBNext FFPE DNA Repair Mix (M6630) and the NEBNext Ultra II End repair/dA-tailing Module (E7546). The adaptors were ligated using the NEBNext Quick Ligation module (E6056) (New England Biolabs, Ipswich, Massachusetts, USA) and purified using AMPure XP beads (Beckman Coulter, Brea, CA, USA). The libraries were loaded onto FLO-MIN106D (R9) flow cells and sequenced on a MinION sequencing platform. Eight (8) MinION runs were done.

### Basecalling and De-novo genome assembly

Raw ‘fast5’ files from eight MinION runs were used as input to Guppy (ver5.011)^17^ for basecalling using the high accuracy (hac) basecalling configuration. This generated 6,728,728 reads totaling 39,879,979,963 bp with an average length of 5,926.8 bp.

**Fig. 2:**
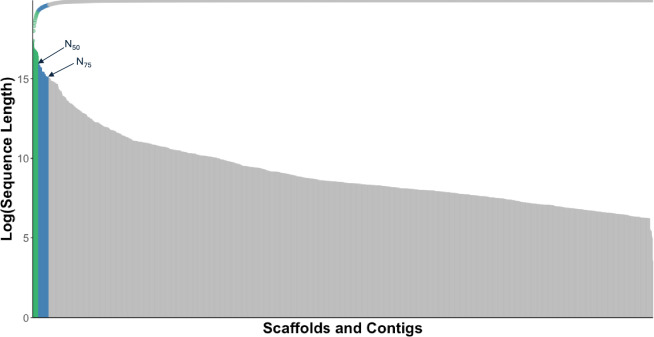
Sequence length distribution: The length distribution of scaffolds and contigs from the Dovyalis assembly on a logarithmic-scale. The scaffolds/contigs above the N50 and N75 are coloured in green and blue respectively.

Two *de novo* draft genome assemblies were generated with the ONT long reads generated above using two long read assemblers, Flye (ver2.8.1)^18^ and wtdbg2 (Redbean) ver2.4^19^, both with default parameters. The assembly generated by Flye was more contiguous, (N50 of 13,328,472 bp, Table 1, Fig. 2) compared to wtdbg2’s assembly (N50 of 1,206,481 bp, Table 1). We therefore retained the Flye assembly for further analyses.

**Table 1:** *Dovyalis afra* Flye and Redbean *de novo* assemblies’ statistics.

Assembly Metric	Flye	wtdbg2 (Redbean)
# contigs (> = 1000 bp)	1,024	4,734
Total length (bp)	440,644,116	449,818,989
GC (%)	33	34
N50 (bp)	13,328,472	1,206,481
N75 (bp)	3,352,526	231,360
L50 (# contigs)	11	74
L75 (# contigs)	32	290
Largest contig (bp)	34,763,902	11,627,461

### RNA extraction and sequencing

Total RNA was extracted from seven tissues representing various developmental stages of the Bark, stem and leaves using the PureLink RNA Mini Kit (Thermo Fisher Scientific, Carlsbad, CA, USA) according to the manufacturer’s instructions. Briefly, 250 mg of fresh tissue was ground in liquid nitrogen using chilled mortar and pestle and homogenized in 1.5 mL of lysis buffer prepared with 2-mercaptoethanol. After brief incubation and centrifugation, 0.5 mL of the lysate was recovered and 0.5 volumes of 96% ethanol added, mixed and passed through an RNA binding column. On-column DNase treatment was done using Purelink DNase and the RNA recovered in 100 μL of RNase free water. RNA yield and quality was checked using Qubit 2.0 (Life Technologies, Carlsbad, CA, USA) and bioanalyzer 2100 (Agilent technologies, Santa Clara, CA, USA). RNA libraries were constructed following the TruSeq RNA Sample Preparation Kit (Illumina, San Diego, CA, USA) manual, and sequenced on the Illumina HiSeq. 2500 platform (paired-end, 100-bp reads), generating about 52 Gbp of sequence data.

### *De novo* transcriptome assembly

A three-step RNAseq data preparation guideline was used to improve its quality for annotation. First, random errors from the RNAseq reads were removed using RCorrector (ver1.0.4)^20^. Secondly, uncorrectable read pairs were removed using FilterUncorrectabledPEfastq.py python script^21^. Lastly, the reads were further processed for adapter removal and quality trimming using TrimGalore (ver 0.6.7)^22^ and the reads quality was assessed using Fastqc (ver 0.11.7)^23^. The clean reads were assembled with Trinity (ver 2.13.2)^24^ using default options. Finally, TransDecoder (ver5.5.0)^25^ was used to predict and identify 77,660 candidate coding regions within the Trinity generated transcripts sequences.

### Repeat annotation

Repetitive regions of the Dovyalis draft assembly were identified using RepeatModeler (ver 1.0.8)^26^ which created a de novo repeat library. The repeat library was used in RepeatMasker (ver 4.0.6)^27^ to identify and classify the repeats in the Dovyalis assembly. Overall, a total of 620,527 interspersed repeats comprising retroelements, DNA transposons, rolling-circles and unclassified repeats totaling 60.42% of the Dovyalis assembly were identified (Table 2).

**Table 2:** The number, type and proportion of interspaced repeat elements identified in the *Dovyalis afra* assembly.

Name	Number of TEs	Length (bp)	% of Assembly
**Retroelements**	80314	94691748	21.49
LINEs:	6113	5505821	1.25
RTE/Bov-B	249	25166	0.01
L1/CIN4	5864	5480655	1.24
LTR elements	74201	89185927	20.24
Ty1/Copia	32649	44785574	10.16
Gypsy/DIRS1	39653	42808137	9.71
**DNA transposons**	12328	11715346	2.66
hobo-Activator	2130	2014028	0.46
Tc1-IS630-Pogo	778	93577	0.02
Tourist/Harbinger	2178	773458	0.18
**Rolling-circles**	1303	1060265	0.24
**Unclassified**	362767	159843511	36.27
**Total**	**620527**	**266250605**	**60.42**

### Gene prediction and functional annotation

The funannotate (ver1.8.15) pipeline^28^ was used for predicting gene models in the repeat-masked Dovyalis genome. The pipeline comprises of four main scripts: “funannotate train” for training the ab-initio gene predictors, “funannotate predict” for predicting gene models, “funannotate update” for refining gene models including of untranslated regions, and “funannotate annotate” for functional annotation of identified gene models. The funannotate pipeline integrates different sources of evidence for predicting gene models including homology (protein evidence), transcripts (expression evidence) and ab-initio predictors.

For homology evidence, we downloaded and combined protein sequences of four well annotated closely related genomes; *Populus trichocarpa* (cottonwood, Ptrichocarpa_v4.1), *Populus deltoides (*eastern cottonwood, Pdeltoides_v2.1), *Salix Purpurea* (purple osier willow, Spurpurea_v5.1) and *Eucalyptus grandis (*flooded gum eucalyptus, Egrandis_v2.0) from Phytozome^29^. Protein sequences of the longest transcripts were used. For ab-initio evidence, the “funannotate train” script was used to train ab-initio gene predictors: Augustus (ver3.3.3)^30^, SNAP (ver2006-07-28)^31^, GlimmerHMM (ver3.0.4)^32^. For this, cleaned RNAseq reads were aligned to the repeat-masked and indexed Dovyalis genome using Hisat2(ver 2.1.0)^33^ and the read alignments were used as input for genome-guided transcript assembly using Trinity^24^ which generated 116,867 transcripts. The transcripts were further aligned back to the genome using PASA (ver2.4.1)^34^ to define complete gene models which were subsequently used to train Augustus, SNAP, GlimmerHMM. Additionally, the de-novo transcript assembly previously described was used as transcript evidence.

Using all this evidence, the “funannotate predict” script generated weighted consensus gene structures using EvidenceModeler^35^. We predicted 27,449 protein-coding gene models and 501 tRNA-coding genes.

Functional annotation of the predicted genes was conducted with ‘funannotate annotate’ script which is based on domain conservation and sequence similarity by searching the predicted sequences against public databases. Here, Interproscan (ver5.25-64.0)^36^ was used to align against InterPro^37^ protein databases; TIGRFAM, SUPERFAMILY, PANTHER, Pfam, PRINTS and ProDom^38^. The gene models were further screened for homology using eggnog-mapper (ver2.1.9)^39^ and parsed the predicted results from Interproscan and eggnog-mapper to Funannotate “annotate” to annotate the putative functions of the protein sequences using CAZYmes^40^, MEROPS^41^, BUSCO^42^, UNIProtKB^43^, PFAM^44^, dbCAN^45^ and GO^46^ Ontologies databases.

## Data Records

The raw reads from ONT DNA sequencing are available on SRA repository under the accession number SRX26387751^47^. Illumina RNASeq as well as the genome assembly and annotation of Dovyalis are available on ENA sequence repository under the study accession number PRJEB71679^48^. The project is composed of seven Illumina RNA sequencing experiments (ERX11817568, ERX11817572, ERX11819575, ERX11819573, ERX11819577, ERX11819576, ERX11819574). The genome assembly is available under the accession number GCA_963924115.1^49^.

## Technical Validation

**Fig. 3:**
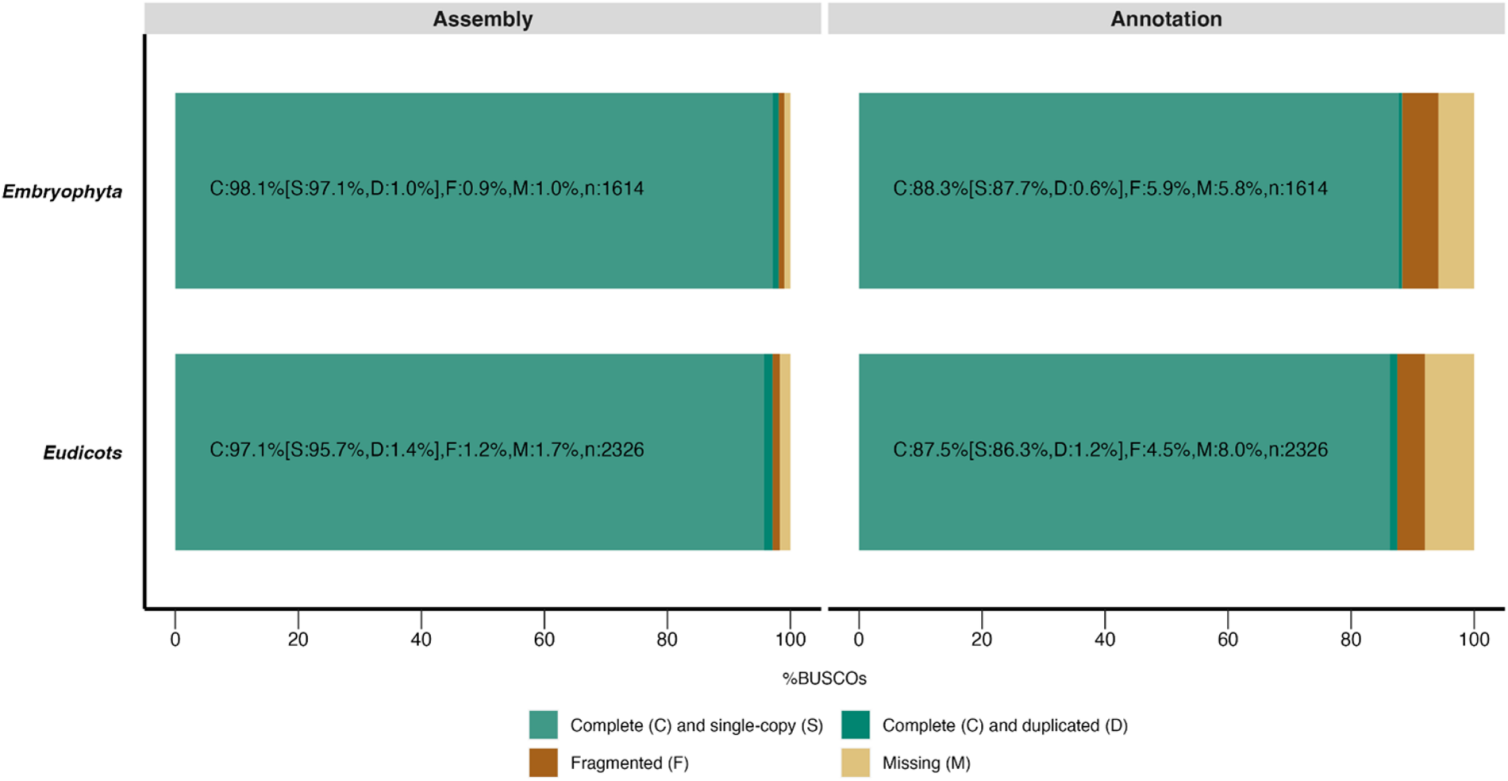
Assessment (BUSCO scores) of the *Dovyalis afra* genome assembly and gene annotation using the Embryophyta and Eudicots reference lineages.

Genome assembly quality was assessed through mapping rate, base accuracy, and completeness. Qualimap (ver2.2.2a)^50^ indicated a high mapping rate of 98.79% for raw ONT reads, with average coverage of 92x, mitigating potential assembly errors associated with Nanopore sequencing. To estimate base accuracy, quality-filtered Illumina RNA-seq reads previously used for transcript evidence in the gene annotation were aligned to the assembly using the splice-aware aligner Tophat2 (ver2.1.0)^51^, and consensus bases generated with SAMtools (ver1.9)^52^. Consensus bases supported by 20–100 Illumina reads exhibited 99.85% accuracy compared to the assembly. Lastly, genome and gene annotation completeness were evaluated using BUSCO (ver5.2.2)^42^. The assembly demonstrated completeness scores of 98.1% and 97.1% against embryophyta_odb10 and eudicots_odb10, respectively, while the gene annotation achieved scores of 88.3% and 87.5%. Both genome and annotation exhibited low duplication and fragmentation rates (<2% and <6%, respectively) (Fig. 3).

## Data Availability

No custom code was used in the work. The analyses were done using open-source bioinformatics software whose versions are indicated in the Methods.
